# Deterministic and Probabilistic Health Risk Assessment of Toxic Metals in the Daily Diets of Residents in Industrial Regions of Northern Ningxia, China

**DOI:** 10.1007/s12011-022-03538-3

**Published:** 2023-01-09

**Authors:** Yan Wang, Deyan Cao, Jiaqi Qin, Siyuan Zhao, Jianzai Lin, Xi Zhang, Junji Wang, Meilin Zhu

**Affiliations:** 1 School of Public Health and Management, Ningxia Medical University, Yinchuan, 750004 China; 2 Key Laboratory of Environmental Factors and Chronic Disease Control, Ningxia Medical University, Yinchuan, 750004 China; 3 College of Basic Medical Sciences, Ningxia medical University, Yinchuan, 750004 China

**Keywords:** Dietary intake, Health risk assessment, Monte Carlo simulation, Toxic metals

## Abstract

This study was designed to investigate the toxic metal (aluminum (Al), arsenic (As), chromium (Cr), cadmium (Cd), copper (Cu), nickel (Ni), lead (Pb), and zinc (Zn)) concentrations in drinking water and different foodstuffs meat (pork, beef, and mutton), cereals (rice, flour, corn, millet), beans (cowpeas, tofu), potatoes (potato, sweet potato), solanaceous fruits (pepper, eggplant, bitter gourd, cucumber), vegetables (cabbage, cauliflower, spinach), and fruits (apples, watermelons, pears, grapes)) and then estimate the potential health risks of toxic metal consumption to local residents in industrial regions of northern Ningxia, China. As in drinking water, Cr in meat, Pb in cereals, Pb in beans, As and Pb in potatoes, Pb in solanaceous fruits, Cr and Ni in vegetables, and Ni and Pb in fruits were the most contaminated heavy metals in the corresponding food with over-standard rates of 16.7%, 12.5%, 5.1%, 60%, 50%, 50%, 38.2%, 44.4%, 44.4%, 31.8%, and 31.8%, respectively.

The results of the deterministic assessment of health risks showed that the total noncarcinogenic risk value of dietary intake of toxic metals by the local population was 5.6106, indicating that toxic metals pose a high noncarcinogenic risk. The order of the non-carcinogenic risk is HI_cereal_ (1.2104) > HI_solanaceous fruit_ (0.9134) > HI_Vegetables_ (0.8726) > HI_Fruit_ (0.8170) > HI_Meat_ (0.7269) > HI_Drinking water_ (0.6139) > HI_Beans_ (0.2991) > HI_Potatoes_ (0.1573). The total carcinogenic health risk from exposure to toxic metals through dietary intake was 9.98 × 10^−4^, indicating that the total cancer risk value of residents is beyond the acceptable range (10^−4^) under the current daily dietary exposure and implies a high risk of cancer. The order of the carcinogenic risk is R_Drinking water_ (2.34 × 10^−4^) > R_Meat_ (2.11 × 10^−4^) > R_solanaceous fruit_ (1.89 × 10^−4^) > R_Fruit_ (1.88 × 10^−4^) > R_cereal_ (1.36 × 10^−4^) > R_Potatoes_ (2.44 × 10^−5^) > R_Vegetables_ (1.51 × 10^−5^) > R_Beans_ (0). The probabilistic assessment results showed that 98.83% of the population is exposed to severe noncarcinogenic risk and 87.02% is exposed to unacceptable carcinogenic risk. The sensitivity analysis showed that drinking water, local cereals, vegetables, and fruits were the major contributors to health risks. Our results indicated that the daily dietary exposure of residents in industrial regions of northern Ningxia poses a serious threat to human health, and it is suggested that relevant departments should strengthen monitoring and control of the current situation of toxic metal pollution in the environment and continue to pay attention and take measures to reduce the exposure of toxic metals in the diets of residents in this area.

**Graphical Abstract:**

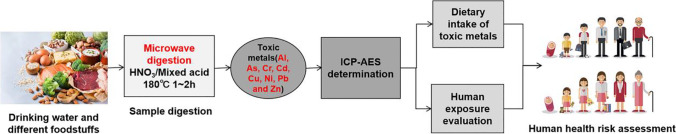

## Introduction

Toxic metal pollution refers to the contamination of the environment by toxic metals or their compounds mainly as a result of human activities, such as mining, waste gas discharge, sewage irrigation, and the use of products containing heavy metals [1, 2]. Toxic metals in the environment are difficult to degrade and are widely distributed in the atmosphere, water, soil, and organisms. Toxic metals are easily absorbed and accumulated by crops and subsequently enter the human body through the food chain. After entering the human body, toxic metals accumulate and can cause acute and chronic damage, in addition to causing potential carcinogenic, teratogenic, and mutagenic hazards. Therefore, toxic metal pollution has raised widespread concern among researchers worldwide [3, 4]. Many studies have investigated toxic metal exposure through the daily diets of residents near industrial parks, with most focusing on economically developed areas, such as smelting and mining areas in Hunan and Guangdong provinces and industrial areas in coastal cities in Zhejiang province, among others. However, few studies have focused on the northern Ningxia region [5–7]. Therefore, it is necessary to investigate the concentrations of toxic metals in the daily diets of residents near the industrial regions of Ningxia.

Health risk assessment refers to the probability estimation of the impact of toxic and harmful substances on human health and safety, which is determined by collecting and analyzing toxicological and epidemiological data, identifying environmental and exposure factors, and other relevant data [8, 9]. The assessment is directly expressed by the degree of health risk, which indicates the possibility of damage to human health [10]. A comprehensive review of the literature has revealed that China’s health risk research is predominantly based on deterministic risk assessment, and probabilistic risk assessment is rarely conducted. However, few studies have used a combination of both approaches to evaluate the exposure levels of toxic metal pollutants in the diets of residents in specific regions [11–13].

Ningxia is located in the inland region of Northwest China in the Yellow River system. Owing to a unique natural environment and resource conditions, several industrial enterprises and industrial parks, including mineral smelting and metal processing, electroplating, electronics, and battery manufacturing industries, were built in northern Ningxia. On the one hand, industrial areas promote economic development; on the other hand, environmental pollution has increased in such areas [14, 15]. In recent years, researchers have observed increased heavy metal pollution in northern Ningxia. Liu et al. [16] analyzed heavy metal pollution in eight categories of foods sold in major supermarkets and farmers’ markets in Ningxia and found that heavy metals, including Pb, Cd, Hg, and Al, were present in the major foods consumed in Ningxia, and their levels exceeded the standard limits to varying degrees. Al had the highest concentration, and its over-standard rate was the highest, indicating the severity of the environmental pollution problem in the area. Therefore, research on Al contamination with regard to food safety risk assessment should be strengthened. Zhao et al. [17] assessed the potential health risks of five heavy metals in water samples obtained from the Qingshui River Basin in Ningxia and observed that the Amaranth River was the most polluted, with the main pollutant being Fe. In addition, the highest total health risk of the five heavy metals was 3.13 × 10^−7^, and Cd, as a carcinogenic pollutant, had the highest health risk index. However, information on the health risk assessment studies of toxic metals through the consumption of foodstuffs is quite limited in the Ningxia industrial region. Moreover, most of the previous studies have only focused on a single or a few kinds of foodstuffs. Thus, a comprehensive risk assessment of toxic metals in the diets of the local residents is urgently needed.

Therefore, the purpose of the present study was to investigate the toxic metal content in the daily diet of residents in the industrial region of Ningxia. In this case, the inductively coupled plasma-atomic emission spectrometry (ICP‒AES) method was used to quantify the heavy metal content in drinking water and various food samples because it has the advantages of a fast analysis speed, high analysis accuracy and precision, and wide determination range. A questionnaire on the dietary consumption of foodstuffs was conducted to obtain region-specific exposure parameters, and the hazard quotient (HQ) and hazard index (HI) were used to assess noncarcinogenic health risks from individual metals and the combined health risk from eight metals, respectively. In addition, the carcinogenic risk (R) was used to assess the carcinogenic risk of As in the diet of local residents. Deterministic and probabilistic risk assessment methods were used to evaluate potential health risks to local residents following dietary exposure to selected toxic metals. A sensitivity analysis was performed to identify the major contributors to the health risks.

## Materials and Methods

### Sample Collection and Preparation

Drinking water and various food samples were collected from nearby villages and towns (38°58′ to 39°54′N, 116°23′ to 106°35′E) in the industrial regions of northern Ningxia, China, in September 2017. Samples were taken after obtaining the relevant permission from families. The 187 samples included 36 drinking water samples and 151 food samples. The 151 food samples included 8 portions of meat (pork, beef, and mutton), 59 portions of cereals (rice, flour, corn, millet), 10 portions of beans (cowpeas, tofu), 4 portions of potatoes (potato, sweet potato), 34 portions of solanaceous fruits (pepper, eggplant, bitter gourd, cucumber), 18 portions of vegetables (cabbage, cauliflower, spinach), and 22 portions of fruits (apples, watermelons, pears, grapes). Five food samples were collected at each site, with every five replicates forming a large sample. Samples of drinking water were placed in plastic bottles. The food samples were dried in a 60 °C oven (DHG-9030A, thermostatic air drying oven, China) to remove moisture. Then the dried samples were crushed and ground, packed into polyethylene bags, and immediately transported to the laboratory for Al, As, Cr, Cd, Cu, Ni, Pb, and Zn analysis.

### Laboratory Analyses of Toxic Metals in Water and Food Samples

Twenty milliliters of each water sample were transferred into a test tube. All samples were initially evaporated in an ED54-iTouch high-temperature digestion furnace (LaiBoT, China), and 5 mL (100%) concentrated nitric acid (HNO_3_) was added to the evaporated samples. Afterward, the samples were digested in a high-temperature digestion furnace. Using a three-step temperature procedure, under the condition of the maximum power of the high-temperature digestion furnace, first, the temperature was increased linearly to 6 ℃ for 20 min; second, the temperature was increased to 120 ℃ and maintained for 30 min; and finally, the temperature was increased to 180 ℃ for 1~2 h. The digested samples were dissolved in deionized water and diluted to 10 mL.

Samples of meat, vegetables, and fruits (0.5000 g) were weighed in test tubes, and 20 mL of mixed acid (v:v, HNO_3_:HClO_4_ = 4:1) was added to each sample. After 1 day of cold digestion, the test tubes were placed in a high-temperature digester for heat digestion. The digested samples were subsequently dissolved in deionized water and diluted to 10 mL.

Cereal samples were digested with 10 mL HNO3, and the rest of the steps were the same as those for the other food samples.

All samples used in the present study were analyzed using inductively coupled plasma-atomic emission spectrometry to determine their Al, As, Cr, Cd, Cu, Ni, Pb, and Zn concentrations. Blank reagents and multielement standard solutions were used for each batch. The accuracy of the methods was validated by three replicate measurements. Quality control was performed to ensure the precision and accuracy of the experiment. The correlation coefficients of the standard curves of the elements were better than 0.9990. Recovery analysis was performed using standard reference plant materials (GBW10014) (from the National Research Center for Standards in China) to ensure the reliability of sample analysis. The recoveries of the elements ranged from 85 to 110%. Each sample was analyzed three times, with relative standard deviations (RSDs) of the repeated analyses below 5%, indicating that the precision and accuracy of the experiment met the requirements.

### Collection of Data on the Diets of Residents in Ningxia Villages and Towns Using Questionnaires

A questionnaire-based survey of village residents was conducted during sampling. The food frequency method was used to design the questionnaires used in the present study. Considering the age distribution and gender balance of the population, a total of 97 completed questionnaires were judged to be effective. Information, including food types, food consumption frequency, food source, age, gender, and the number of household members, was recorded.

### Health Risk Assessment

#### Deterministic Estimation of Health Risks

Of the eight toxic metals studied, Al, Cr, Cd, Cu, Ni, Pb, and Zn posed noncarcinogenic health risks through oral exposure, whereas As posed both noncarcinogenic and carcinogenic health risks through oral exposure. The daily exposure to toxic metals was calculated using Eqs. 1 and 2 as follows [18, 19]:

1 $${EXPO}_{\mathrm{As}}=\frac{\mathrm{C }\times \mathrm{ DI }\times \mathrm{ EF }\times \mathrm{ ED}}{\mathrm{BW }\times \mathrm{ LT}}$$

2 $$EXPO=\frac{\mathrm{C}\times \mathrm{DI }\times \mathrm{ EF }\times \mathrm{ ED}}{\mathrm{BW }\times \mathrm{ LT}}$$

where EXPO is the daily exposure to toxic metals; C (mg/kg) is the concentration of toxic metals in drinking water and foods; DI (g/day) is the daily intake of different types of food; EF (day/year) is the exposure frequency, which was obtained from the questionnaire; ED (years) is the duration of exposure; BW (kg) is the average body weight of the resident, which was also determined from the questionnaire; and LT is the average exposure time of the resident, which was assumed to be 70 years [20].

The hazard quotient (HQ) was calculated to assess the noncarcinogenic risk of individual toxic metals. An HQ ≥ 1 indicates that the risk is serious, whereas an HQ < 1 indicates that it is not serious. HQ was calculated using Eq. 3 as follows [21, 22]:

3 $$HQ=\frac{\mathrm{EXPO}}{\mathrm{RfD}}$$

where RfD (mg/(kg·d)) is the reference dose of noncarcinogenic pollutants, as recommended by the United States Environmental Protection Agency (US EPA) and the World Health Organization (WHO) [23]. The RfD values are listed in Table 1.

**Table 1:** Reference dose values of various heavy metals in drinking water and food

	Drinking water	Food
Al	1.0000	0.0005
As	0.0003	0.0003
Cr	0.0030	0.0030
Cd	0.0050	0.0010
Cu	0.0400	0.0370
Ni	0.0200	0.0200
Pb	0.0014	0.0037
Zn	0.3000	0.3000

When exposure to multiple noncarcinogenic substances occurs simultaneously, regardless of their interactions, an overall hazard index (HI) can be obtained by summing the cumulative noncarcinogenic risk of each pollutant. The HI was calculated using Eq. 4 and used to assess the total noncarcinogenic health risk: an HI value ≤ 1 means that the exposed population is unlikely to experience notable adverse effects, and if the value is > 1, there may be noncarcinogenic effects on human health [24, 25].

4 $$HI={\sum }_{1}^{\mathrm{n}}{\mathrm{HQ}}_{\mathrm{n}}$$

This index is used for the total risk caused by several constituents in a material or the sum of more than one HQ for a specific constituent that enters the body through various pathways. In this study, HI was used to assess the cumulative health risk to humans from exposure to eight toxic metals in food.

The metal As is a carcinogen, and its carcinogenic risk (R) can be calculated using Eq. 5 as follows [26]:

5 $$R=\mathrm{SF}\times \mathrm{EXPO}$$

where SF is the carcinogenic risk slope factor [mg/(kg·d)], and the US EPA recommends an SF of 1.5 [mg/(kg·d)] for As. The negligible carcinogenic risk level recommended by the US EPA is 10^−6^, while the level recommended by the WHO is 10^−5^; the maximum acceptable level recommended by the US EPA is 10^−4^ [27, 28].

#### Probabilistic Assessment and Sensitivity Analysis

Uncertainty analysis in health risk assessment mainly includes two components: determining the probabilistic results and evaluating the contribution of each variable to the results. Monte Carlo simulation was used to analyze the uncertainty of the results [29]. First, the best-fitting probability distribution type of the exposure factors was simulated by Anderson-Darling and chi-square tests. Subsequently, stable exposure distribution results were obtained from 10,000 iterations, and the values at different quantiles (e.g., 10th, 50th, and 90th) of the exposure distribution results were used to assess the probabilistic risk. The degrees of contribution of the exposure factors to the results were assessed by performing sensitivity analysis. A positive value indicated that the exposure factor was positively correlated with health risks; otherwise, it was negatively correlated [30–32].

#### Statistical Analysis

The mean, standard deviation (SD), and over-standard rates were calculated using MS Excel 2010 (Microsoft Corp, Redmond, WA, USA). The determination of the best-fitting distribution for each parameter, Monte Carlo simulation, and sensitivity analysis were performed using the Crystal Ball.

#### Results and Discussion

### Heavy Metal Concentrations in Drinking Water and Various Food Samples

The average concentrations of the eight toxic metals (Al, As, Cr, Cd, Cu, Ni, Pb, and Zn) and their corresponding SD values are listed in Table 2. In the present study, the over-standard rates of Al, As, Ni, and Pb in drinking water samples were 11.1%, 16.7%, 5.6%, and 11.1%, respectively, and As exhibited the highest level of pollution. The ingestion of trace amounts of As over a long period may be detrimental to human health because of its non-biodegradable nature [33]. Ahmed et al. [34] reported high concentrations of As in the transboundary Langat River in Malaysia. Therefore, it is important to manage drinking water sources to minimize human health risks associated with As exposure.

**Table 2:** Concentrations of heavy metals in drinking water and food

		Al	As	Cr	Cd	Cu	Ni	Pb	Zn
Drinking water *n* = 36(mg/L)	Mean	0.0997	0.0056	0.0005	0.0000	0.0012	0.0054	0.0037	0.0296
	SD	0.1342	0.0111	0.0006	0.0001	0.0024	0.0226	0.0040	0.0549
	Mix	0.0000	0.0000	0.0000	0.0000	0.0000	0.0000	0.0000	0.0000
	Max	0.5429	0.0456	0.0022	0.0004	0.0120	0.1361	0.0121	0.2372
	Safe limits	0.2	0.01	0.05	0.005	1.0	0.02	0.01	1.0
	Over-limit ratio	11.1%	16.7%	0%	0%	0%	5.6 %	11.1%	0%
Meat *n* = 8(mg/Kg)	Mean	0.0202	0.2183	0.4719	0.0000	1.0747	0.0000	0.0780	46.3887
	SD	0.0533	0.1581	0.4061	0.0000	0.5379	0.0000	0.0816	19.4477
	Mix	0.0000	0.0000	0.0000	0.0000	0.4116	0.0000	0.0000	15.4314
	Max	0.1613	0.4269	1.0140	0.0000	2.2011	0.0000	0.1960	73.5856
	Safe limits	10	0.5	1.0	0.1	10	0.2	0.2	100
	Over-limit ratio	0%	0%	12.5%	0%	0%	0%	0%	0%
Cereal *n* = 59(mg/Kg)	Mean	0.0055	0.0158	0.2564	0.0128	0.2820	0.1125	0.0453	5.3166
	SD	0.0388	0.0276	0.2363	0.0142	0.2017	0.1987	0.0745	3.5065
	Min	0.0000	0.0000	0.0000	0.0000	0.0000	0.0000	0.0000	0.0000
	Max	0.3005	0.0989	0.7701	0.0656	0.6861	1.2914	0.2835	12.1507
	Safe limits	100	0.5	1.0	0.1	10	0.4	0.2	50
	Over-limit ratio	0%	0%	0%	0%	0%	1.7%	5.1 %	0%
Beans *n* = 10(mg/Kg)	Mean	1.7096	0.0000	0.7536	0.0045	1.9198	0.4792	0.4636	8.5279
	SD	1.1480	0.0000	0.8636	0.0091	1.7087	0.6002	0.3877	7.1609
	Min	0.3980	0.0000	0.0550	0.0000	0.4282	0.0000	0.0000	1.7347
	Max	4.1777	0.0000	2.2808	0.0301	4.6906	1.6047	1.0014	22.1708
	Safe limits	100	0.5	1.0	0.2	20	3.0	0.2	100
	Over-limit ratio	0%	0%	40%	0%	0%	0%	60%	0%
Potatoes *n* = 4(mg/Kg)	Mean	0.4319	0.1475	0.0322	0.0000	0.1316	0.1579	0.1798	0.2276
	SD	0.1784	0.1475	0.0187	0.0000	0.0674	0.0683	0.1894	0.3941
	Mix	0.1349	0.0000	0.0155	0.0000	0.0287	0.0895	0.0000	0.0000
	Max	0.5866	0.2984	0.0611	0.0000	0.2097	0.2639	0.4436	0.9102
	Safe limits	10	0.2	0.5	0.1	6	0.3	0.2	5
	Over-limit ratio	0%	50%	0%	0%	0%	0%	50%	0%
Solanaceous fruit *n* = 34(mg/Kg)	Mean	0.0872	0.0965	0.2728	0.0185	0.8822	0.1461	0.1659	5.3943
	SD	0.1180	0.1237	0.2676	0.0262	0.8868	0.1732	0.2390	4.3754
	Mix	0.0000	0.0000	0.0000	0.0000	0.0885	0.0000	0.0000	0.0000
	Max	0.3590	0.3722	0.9064	0.1096	3.3155	0.6618	0.8709	15.8353
	Safe limits	10	0.5	0.5	0.05	10	0.3	0.1	20
	Over-limit ratio	0%	0%	26.5%	17.6%	0%	11.8%	38.2%	0%
Vegetables *n* = 18(mg/Kg)	Mean	0.0064	0.0042	0.5642	0.0277	0.9527	0.5560	0.1603	6.7956
	SD	0.0143	0.0173	0.4771	0.0237	0.9718	0.9483	0.4142	5.5740
	Mix	0.0000	0.0000	0.0440	0.0000	0.0197	0.0000	0.0000	1.3239
	Max	0.0549	0.0753	1.5511	0.0722	2.8231	3.4651	1.8107	16.6289
	Safe limits	10	0.5	0.5	0.2	10	0.3	0.3	20
	Over-limit ratio	0%	0%	44.4%	0%	0%	44.4%	11.1%	0%
Fruit *n* = 22(mg/Kg)	Mean	0.0171	0.0473	0.0673	0.0173	0.3318	0.2458	0.1496	4.4962
	SD	0.0555	0.1438	0.0303	0.0210	0.2118	0.3922	0.2136	10.6937
	Mix	0.0000	0.0000	0.0093	0.0000	0.1261	0.0000	0.0000	0.0000
	Max	0.2420	0.6893	0.1172	0.0759	1.0795	1.5844	0.5846	50.6054
	Safe limits	15	0.5	0.5	0.05	10	0.2	0.1	5
	Over-limit ratio	0%	4.5%	0%	4.5%	0%	31.8%	31.8%	13.6%

Excessive amounts of Cr were detected in meat samples, with the metal content exceeding the recommended limit by 12.5%. Previous studies have also reported that Cr concentrations in commercial livestock meat exceeded the standard value, although the over-standard rates of Cr varied among different livestock meats. Animals ingest heavy metals that are added to their feed, which once in the body are difficult to remove.

Our results showed that the Pb and Ni contents in cereal samples exceeded the standard limits by 5.1% and 1.7%, respectively. Nawaz et al. [37] investigated Ni accumulation in cereals irrigated with wastewater and observed that high concentrations of irrigation wastewater improved the bioavailability of Ni in crops and in turn increased its accumulation in the edible parts of crops. Román-Ochoa et al. [38] examined the heavy metal content of cereals and grain-processed foods in Arequipa, Peru, and found that lead concentrations in quinoa, corn, and rice products increased sequentially due to processing. Therefore, the high Pb and Ni concentrations in cereals should be addressed, and their monitoring should be enhanced.

According to the results of the present study, the Cr and Pb concentrations in soybean were high, and their over-standard rates were 40% and 60%, respectively. These results are consistent with those obtained by Huang et al. [39], who reported that the Cr and Pb concentrations in soybean were 0.03–1.05 mg/kg and 0.11–0.85 mg/kg, respectively, and the Pb concentration was high, with an over-standard rate of 97.4%. The As and Pb concentrations in potato samples exceeded the standard limits, and their over-standard rate was 50%. Tubers, such as potatoes, are prone to heavy metal contamination. Furthermore, tubers are the most heavily metal-enriched plant parts. Bao et al. [40] assessed the heavy metal contents of potatoes planted in an experimental area of the Qinghai University Academy of Agriculture and Forestry Sciences, and the results showed that the respective average contents of Cd, Cr, Pb, and Ni in the potato tubers were 3.20-fold, 1.58-fold, 9.4-fold, and 1.7-fold higher than the limit values of the metal contents in potato tubers. Bao et al. observed that the Pb content exceeded the standard limit and exhibited the highest level of pollution, which is consistent with the results of the present study.

The over-standard rates of Cr, Cd, Ni, and Pb in solanaceous fruit samples were 26.5%, 17.6%, 11.8%, and 38.2%, respectively. The order of heavy metal contamination in solanaceous fruits from the highest to the lowest was Pb > Cr > Cd > Ni. However, vegetables are easily contaminated by heavy metals, such as Pb and Cd, and the phenomenon of exceeding their standard limits is common. Luo et al. [41] investigated the health risks of heavy metals in solanaceous fruits in Beijing, China. The results showed that the average Pb, Cd, Cu, and Zn contents in solanaceous fruits in Beijing were 0.082, 0.021, 0.996, and 0.590 mg/kg, respectively. Based on the national food safety standard limits of China, the over-standard rate of Pb in solanaceous fruits in Beijing was 5.8%, and its pollution level was the highest. The results obtained by Luo et al. are similar to those of the present study.

The over-standard rates of Cr, Ni, and Pb in vegetable samples were 44.4%, 44.4%, and 11.1%, respectively. The results showed that the pollution levels of Cd, Ni, and Zn in leafy vegetables were relatively high. Frazana et al. [42] reported results similar to those of this study. The report stated that heavy metal concentrations were measured in vegetables commonly eaten in industrial areas of Bangladesh. Pb, Cd, Cr, and Ni were detected in most of the vegetables analyzed, and some samples had Pb, Cd, and Ni concentrations that exceeded the FAO/WHO maximum permissible concentrations for Pb, Cd, and Ni. Previous studies have shown that the accumulation capacity of heavy metals in vegetables is usually ranked as vegetables > legumes > solanaceous fruits. The variations in the distribution of heavy metal pollutants in various types of vegetables were associated with the differences in the biological characteristics of the vegetables.

Our results showed that As, Cd, Ni, Pb, and Zn were detected in the fruit samples, and their concentrations were higher than those reported in related studies, suggesting that the fruits in the northern region of Ningxia are highly polluted with heavy metals. Further analysis suggested that the cause of the over-standard concentrations could be severe heavy metal pollution in soils adjacent to the industrial park, where most of the fruits are planted by the local residents.

The heavy metal concentrations in the drinking water and various foods analyzed in the present study were observed to have the following order: drinking water (Al > Zn > As > Ni > Pb > Cu > Cr > Cd), meat (Zn > Cu > Cr > As > Pb > Al > Ni = Cd), cereals (Zn > Cu > Cr > Ni > Pb > As > Cd > Al), beans (Zn > Cu > Al > Cr > Ni > Pb > Cd > As), potatoes (Al > Zn > Pb > Ni > As > Cu > Cr > Cd), solanaceous fruits (Zn > Cu > Cr > Pb > Ni > As > Al > Cd), vegetables (Zn > Cu > Cr > Ni > Pb > Cd > Al > As), and fruits (Zn > Cu > Ni > Pb > Cr > As > Cd > Al). A summary of the concentrations of heavy metals in drinking water from different countries is presented in Table 3, whereas a summary of heavy metals in various types of food is presented in Table 4.

**Table 3:** A summary of the concentrations of heavy metals in drinking water from different countries

Concentration of metal (ug/L) in the drinking water								Country	Author and year of publication
Al	As	Cr	Cd	Cu	Ni	Pb	Zn		
		1.6	0.1		1.6	0.9		India [45]	Prasad M, et al. (2022)
	2.185		0.002			0.11		Thailand [46]	Wongsasuluk P, et al. (2018)
		0.37	0.42					Malaysia [47]	Ahmed MF, Mokhtar MB. (2020)
	0.18	4.49			1.69	0.58		Iran [48]	Alidadi H, et al. (2019)
	0.97	0.48		0.1	0.36	0.1	4.26	China [49]	Yan M, et al. (2018)
		15.0	1.25	515.0	17.0	8.0	785.0	Ethiopia [50]	Haftu Z, et al. (2020)
	0.35	4.13	0.03	16.10	1.10	2.03		Pakistan，Swabi [51]	Hussain S, et al. (2019)
	< 1		< 1	< 1	2.0	< 1	20.0	China, Beijing [23]	Xie, et al. (2021)
			10-20			50-70		South Africa [52]	Madilonga RT, et al. (2021)
			3.79	4.74		11.02	7.17	Africa [53]	Ali S, et al. (2022)

**Table 4:** A summary of the concentrations of heavy metals in various types of food

Type of food	Concentration of metal (mg/Kg)								Country	Author and year of publication
	Al	As	Cr	Cd	Cu	Ni	Pb	Zn		
Meat										
Baked ham			0.15	0.01	1.08		0.22	6.02	Italy [54]	Barone G, et al. (2021)
Raw ham			0.19	0.01	1.11		0.30	5.71		
Mortadella			0.20	0.02	1.13		0.34	6.03		
Meat			0.87		0.95			43.30	Spain [55]	Perelló G, et al. (2015)
Meat			0.019	0.002	0.74		0.004	22.50	Swedish [56]	Becker W, et al. (2011)
Meat			0.21	0.004					India [57]	Dubey VK, et al. (2016)
Beef			1.24			0.25		121.27	Nigeria [58]	Ihedioha JN, et al. (2014)
Fresh meat		0.018	0.061	0.002	0.801	0.055	0.029		China [59]	Han JL, et al. (2022)
Mutton		349	362	0.301			0.482		Kuwait [60]	Abd-Elghany SM, et al. (2020)
Sheep		0.23	1.66	2.79	5.56	0.54	11.79	233.10	Iran [61]	Raeeszadeh M, et al. (2022)
Beef		0.2	1.83	4.31	5.75	1.17	7.01	234.6		
Cereal, beans, and potatoes										
Wheat		0.111	0.094	0.016	2.640	0.275	0.044	21.528	Iran [62]	Ghanati K, et al. (2019)
Flour		0.004		0.043			0.067		Poland [63]	Bielecka J, et al. (2022)
Rice			0.41	0.13		0.84	< 0.05		Nigeria [64]	Orisakwe OE, et al. (2015)
Wheat			0.42	0.16		0.41	< 0.05			
Grain		0.028		0.018			0.103		China [65]	Liu, et al. (2019)
Grain		0.087	0.082	0.001	0.032		0.013		Malaysia [66]	Zulkafflee NS, et al. (2022)
Rice			1.98	0.08		1.50	0.15	13.92	Burkina Faso [67]	Bazié BSR, et al. (2022)
Maize		0.53	1.2	0.12		0.080	0.46		Bangladesh [68]	Rahman M, et al. (2019)
Pea		0.61	0.64	0.23		0.60	0.97			
Beans		0.042	0.120	0.025	1.466		0.117	4.363	China [69]	Qi, et al. (2022)
Beans		< dl	< dl	0.04	9.2	2.45	0.25	72.0	Croatia [70]	Stančić Z, et al. (2016)
White potato		0.027	< dl	0.23	8.2	0.31	0.36	57.4		
Red potato		0.053	< dl	0.28	5.9	< dl	0.17	60.4		
Potato			1.13	0.6	4.8	0.89	3.04	37.66	Pakistan [71]	Din AU ,et al. (2013)
potato		0.448		0.135	0.015		0.613	4.674	China [72]	Zhou, et al. (2016)
Vegetable and fruit										
Cabbage			4.33	1.79			7.14		Iran [73]	Jafarian-Dehkordi A, et al. (2013)
Lettuce			6.00	1.79			5.16			
Carrot		1.82	8.80	0.15	6.80	0.00	6.96	2.87	Colombia [74]	Lizarazo MF, et al. (2020)
Mushroom		2.58	12.8	0.16	19.4	0.00	5.69	3.31		
Spinach					29.65		14.61	137.84	India [75]	Sonu K, et al. (2019)
Cabbage					6.30		24.2	80.02		
Vegetable		0.025	0.127	0.140	0.600		0.171	3.725	China [69]	Qi, et al. (2022)
Solanaceous vegetables		0.019	0.083	0.031	0.546		0.058	2.101		
Cabbage		6.36	4.50	1.00	15.96	3.06	6.70	34.4	Ethiopia [76]	Bayissa LD, et al. (2021)
Grapes			1.06		3.21	0.31			Egypt [77]	Amer MM, et al. (2019)
Apple		0.003		0.002			0.068		India [78]	Mawari G, et al. (2022)
Fruit					63.8	8.8		47.8	French [79]	Cherfi A, et al. (2016)
Grape			0.002	0.003		0.001	0.04		Bangladesh [80]	Afrin S, et al. (2021)
Apple			0.002	0.001		0.001	0.05			
Orange			0.002	0.001		0.002	0.06			
Strawberry				0.050			0.10		Poland [81]	Rusin M, et al. (2021)
Raspberry				0.050			0.10			
Assembling	8.162	0006	0.286	0.001	0.416		0.041	0.411	China [82]	Nie, et al. (2021)

### Exposure Factors

The daily intake and frequency of exposure to toxic metals through drinking water and various foods are presented in Table 5. The consumption of drinking water, meat, cereals, beans, potatoes, solanaceous fruits, vegetables, and fruit was 1546.39 mL, 42.41 g, 313.92 g, 23.75 g, 17.24 g, 111.28 g, 152.69 g, and 240.59 g, respectively, which satisfied the recommendations of the Dietary Guidelines for Chinese Residents (2022 edition) [83]. The average weight of the participants was 54.93 kg.

**Table 5:** Exposure factors associated with drinking water and various foods

	Daily intake of food (g/day)	Exposure frequency (day/year)	Exposure duration (year)	Average weight (Kg)
Drinking water	1546.39	365	70 [84]	54.93
Meat	42.41	304.09		
Cereal	313.92	365		
Beans	23.75	64.35		
Potatoes	17.24	128.17		
Solanaceous fruit	111.28	235.31		
Vegetables	152.69	316.46		
Fruit	240.59	220.86		
Data sources	Questionnaire	Questionnaire	Document literature	Questionnaire

### Human Health Risk Assessment

#### Determination of the Assessment

The results of the deterministic assessments of the health risks of exposure to toxic metals through drinking water and food are presented in Table 6. For noncarcinogenic risk, an HQ value of individual metals < 1 indicates that the metal poses no significant noncarcinogenic risk through drinking water or various foods. Among the eight toxic metals, Al in beans and potatoes posed higher noncarcinogenic risks than the other metals. The As in drinking water, meat, solanaceous fruits, and fruits posed the highest noncarcinogenic risk. Cr had the highest HQ value in cereals and vegetables. The HI values of drinking water and different foods were as follows: cereals (1.2104) > solanaceous fruits (0.9134) > vegetables (0.8726) > fruits (0.8170) > meat (0.7269) > drinking water (0.6139) > beans (0.2991) > potatoes (0.1573). With regard to the carcinogenic risks posed by As, the order of *R* values was as follows: drinking water (2.34 × 10^−4^) > meat (2.11 × 10^−4^) > solanaceous fruits (1.89 × 10^−4^) > fruits (1.88 × 10^−4^) > cereals (1.36 × 10^−4^) > potatoes (2.44 × 10^−5^) > vegetables (1.51 × 10^−5^) > beans (0.00). The *R* values of drinking water, meat, solanaceous fruits, fruits, and cereals were greater than 1.0 × 10^−4^, indicating that dietary intake poses a significant carcinogenic risk to residents. The *R* values of potatoes and vegetables ranged between 10^−5^ and 10^−4^, indicating that there is a certain level of carcinogenic risk. The *R* value of beans was 0 because As was not detected in beans, and the total carcinogenic risk value attributed to As in the daily diet of residents was greater than 1.0 × 10^−4^, which indicates that local residents may be exposed to potential carcinogenic risks through the diet.

**Table 6:** Noncarcinogenic risk hazard quotients (HQ), toxic metal hazard indices (HI), and carcinogenic risk (R) values of dietary intake of arsenic based on the deterministic estimation method

	Non-carcinogenic risk (HQ)									Carcinogenic risk (R) As
	Al	As	Cr	Cd	Cu	Ni	Pb	Zn	HI	
Drinking water	0.0028	0.5210	0.0045	0.0002	0.0008	0.0076	0.0742	0.0028	0.6139	2.34 × 10^−4^
Meat	0.0259	0.4080	0.1012	0.0000	0.0187	0.0000	0.0136	0.0995	0.7269	2.11 × 10^−4^
Cereal	0.0627	0.3018	0.4884	0.0734	0.0806	0.0321	0.0700	0.1013	1.2104	1.36 × 10^−4^
Beans	0.2621	0.0000	0.0191	0.0003	0.0040	0.0018	0.0096	0.0022	0.2991	0.0000
Potatoes	0.0952	0.0542	0.0012	0.0000	0.0004	0.0009	0.0054	0.0001	0.1573	2.44 × 10^−5^
solanaceous fruit	0.2277	0.4201	0.1187	0.0242	0.0311	0.0095	0.0586	0.0235	0.9134	1.89 × 10^−4^
Vegetables	0.0310	0.0336	0.4532	0.0667	0.0621	0.0670	0.1044	0.0546	0.8726	1.51 × 10^−5^
Fruit	0.0904	0.4180	0.0595	0.0459	0.0238	0.0326	0.1072	0.0397	0.8170	1.88 × 10^−4^

We assessed potential health risks by comparing the HI values of drinking water and various foods, and the results were as follows. The HI value of cereals was > 1, indicating that there may be significant potential health risks associated with the consumption of cereals alone among the local residents in northern Ningxia. Zhang et al. [85] reported that the total target hazard quotient (TTHQ) value of cereals in Binzhou, China, was 0.914, indicating that the combined exposure risk of Pb, Cd, and As intake in cereals was within the acceptable intake levels, although there were potential risks. If multiple heavy metals were monitored, the risk of TTHQ in cereals was greater than one, which verifies the results of the present study. Therefore, the monitoring of multiple heavy metals in cereals should be strengthened, and more comprehensive risk assessments should be carried out. Except for cereals, the HI values for residents who consumed any of the other foodstuffs were generally < 1, indicating that there is no significant potential health risk for residents by consuming individual foodstuffs. The potential health risk of consuming beans or potatoes was the lowest. Xu et al. [86] reported that the health risks of commercially available legumes in Guizhou Province, China, were low and posed no apparent health hazards to humans. Yang et al. [87] reported that local residents in Henan Province, China, were slightly exposed to Pb contamination through grains and potatoes, and the health risks from their exposure were within the acceptable levels. Farzana et al. [42] reported that the Cd levels in all vegetables analyzed in the study were above the threshold for cancer risk and were a potential carcinogenic risk for both adults and children. The noncarcinogenic risk results showed that the THQ values of Pb, Cd, Cr, and Ni in most vegetable samples were all less than 1.0, meaning that there were no harmful health effects on exposed consumers and their health was not compromised. Sadia et al. [80] tested the extent of heavy metal contamination in grapes, apples, oranges, bananas, and pomegranates widely consumed in supermarkets in Dhaka, Bangladesh, to assess their health risk to humans, and the results showed that the lifetime increased risk of cancer from Cd and Pb in bananas, apples, grapes, oranges, and pomegranates for children and adults exceeded the threshold, posing a potential cancer risk to human health, and heavy metal contamination in all types of fruit did not pose a potential noncarcinogenic risk to humans. However, when individual health risks for all toxic metals ingested through drinking water, cereals, meat, vegetables, and fruits were added together, the TTHQ value was 5.6106, suggesting that residents living in northern Ningxia may have adverse health effects. Liang et al. [88] investigated heavy metal pollution and health risks for residents near tailing ponds in Guangdong province in southern China and observed that the total noncarcinogenic HI value of all metals considered through multiple exposure routes was 26.6, which was significantly higher than the acceptable level. In addition, As accounted for the highest cancer risk when cancer risk was considered, and the total cancer risk value was higher than the acceptable range. The results obtained in the present study are consistent with those of Liang et al. Notably, the THQ value is a highly conservative and relative index, and a TTHQ value exceeding one does not imply that local residents have been exposed to adverse health effects. However, according to our results, the potential health risks of As in drinking water were the highest. Therefore, it is necessary to enhance the monitoring of As levels in foodstuffs and issue a consumption advisory note.

#### Probabilistic Assessment

The toxic metal contents in drinking water and various foods analyzed were fitted to lognormal distributions. The DI, EF, and BW values of all the residents were fitted to lognormal distributions. The BWs of males and females were fitted to Poisson and negative binomial distributions, respectively.

**Fig. 1:**
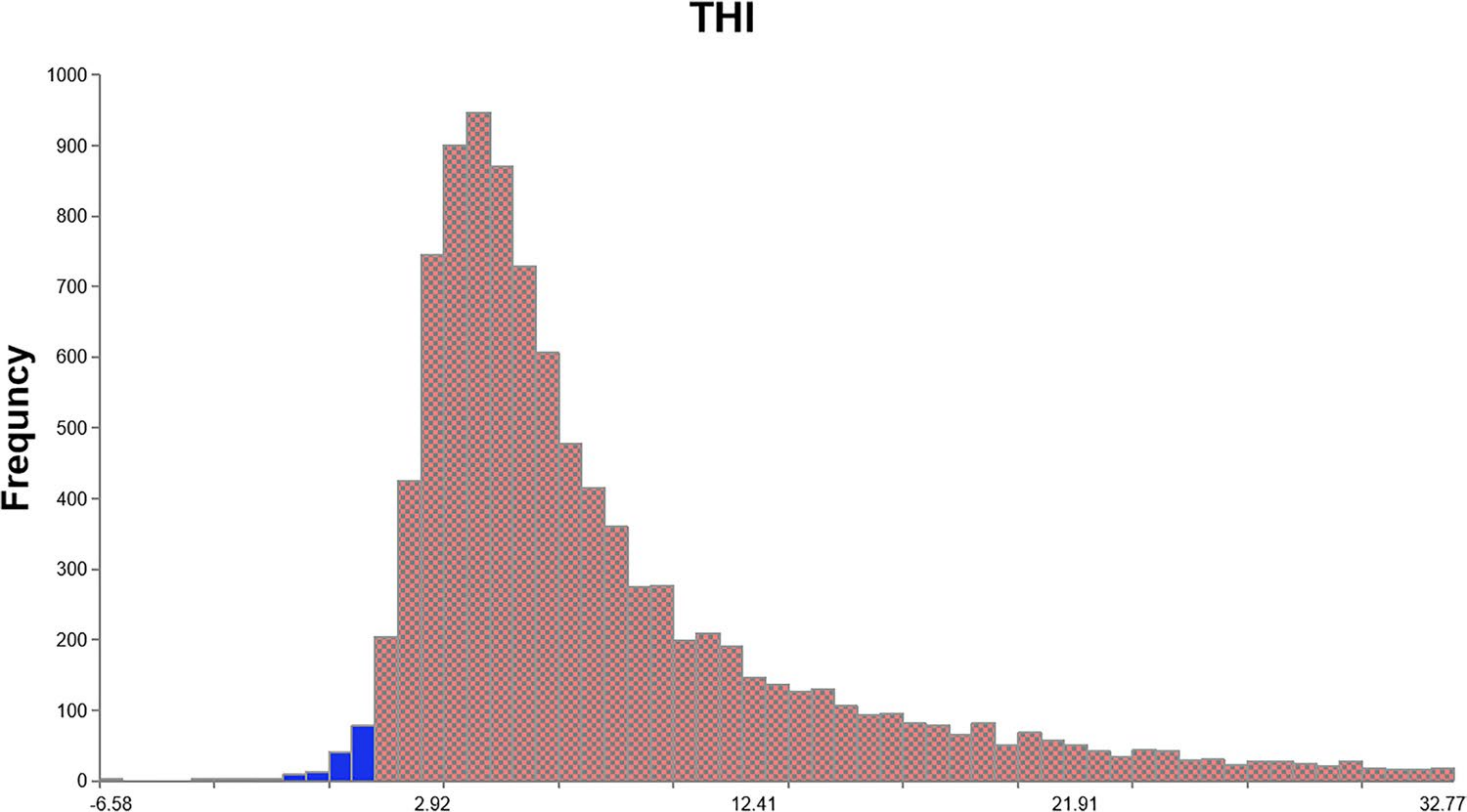
Cumulative distribution of hazard indices for total dietary intake of toxic metals (red area represents the probability of exceeding the maximum acceptable levels, which is 98.83%)

The results of the probabilistic estimation of health risks are summarized in Table 7. All HI values for the noncarcinogenic risk were fitted to lognormal distributions. The 10th percentile of the HI values for drinking water and various foods did not exceed the limit value of 1, indicating that 10% of the residents did not have significant noncarcinogenic risk caused by dietary exposure to the toxic metals. The 50th percentile of the HI values for cereals (1.25) and solanaceous fruits (1.05) were greater than the limit value of 1, indicating that 50% of the residents may have significant noncarcinogenic risks from the consumption of cereals and solanaceous fruits. The 90th percentile of the HI values for drinking water, meat, cereals, solanaceous fruits, vegetables, and fruits were 2.93, 2.39, 4.34, 3.44, 2.98, and 2.62, respectively, indicating that these foods are likely to cause significant health hazards to local residents. The percentage of target hazard index values > 1 (Fig. 1) was 98.83%, indicating that 98.83% of the local residents were exposed to severe noncarcinogenic risk.

**Table 7:** Statistics of probabilistic estimation of hazard indices (HI) and carcinogenic risk (R) values

	Non-carcinogenic risk (HI)					Carcinogenic risk (R) (×10^−4^)				
	Distribution	Parameters	10%	50%	90%	Distribution	Parameters	10%	50%	90%
Drinking water	Lognormal	Location:0.00, Mean:0.95, SD:2.01	0.00	0.65	2.93	Lognormal	Location:0.00, Mean:3.58, SD:8.91	0.00	2.43	12.21
Meat	Lognormal	Location:0.00, Mean:1.14, SD:1.12	0.34	0.77	2.39	Lognormal	Location:0.00, Mean:3.31, SD:3.96	0.71	2.07	7.00
Cereal	Lognormal	Location:0.00, Mean:1.69, SD:2.38	0.00	1.25	4.34	Lognormal	Location:0.00, Mean:1.52, SD:7.52	0.00	1.66	8.56
Beans	Lognormal	Location:0.00, Mean:0.47, SD:0.48	0.12	0.31	0.97	-*	-	-	-	-
Potatoes	Lognormal	Location:0.00, Mean:0.25, SD:0.24	0.08	0.17	0.51	Lognormal	Location:0.00, Mean:0.39, SD:0.64	0.06	0.21	0.86
Solanaceous fruit	Lognormal	Location:0.00, Mean:1.39, SD:1.96	0.00	1.05	3.44	Lognormal	Location:0.00, Mean:2.56, SD:7.09	0.00	2.32	9.15
Vegetables	Lognormal	Location:0.00, Mean:1.39, SD:1.58	0.33	0.88	2.98	Lognormal	Location:0.00, Mean:0.27, SD:1.83	0.00	0.04	0.48
Fruit	Lognormal	Location:0.00, Mean:1.35, SD:9.06	0.22	0.68	2.62	Lognormal	Location:0.00, Mean:3.24, SD:40.50	0.05	0.71	6.21

**Fig. 2:**
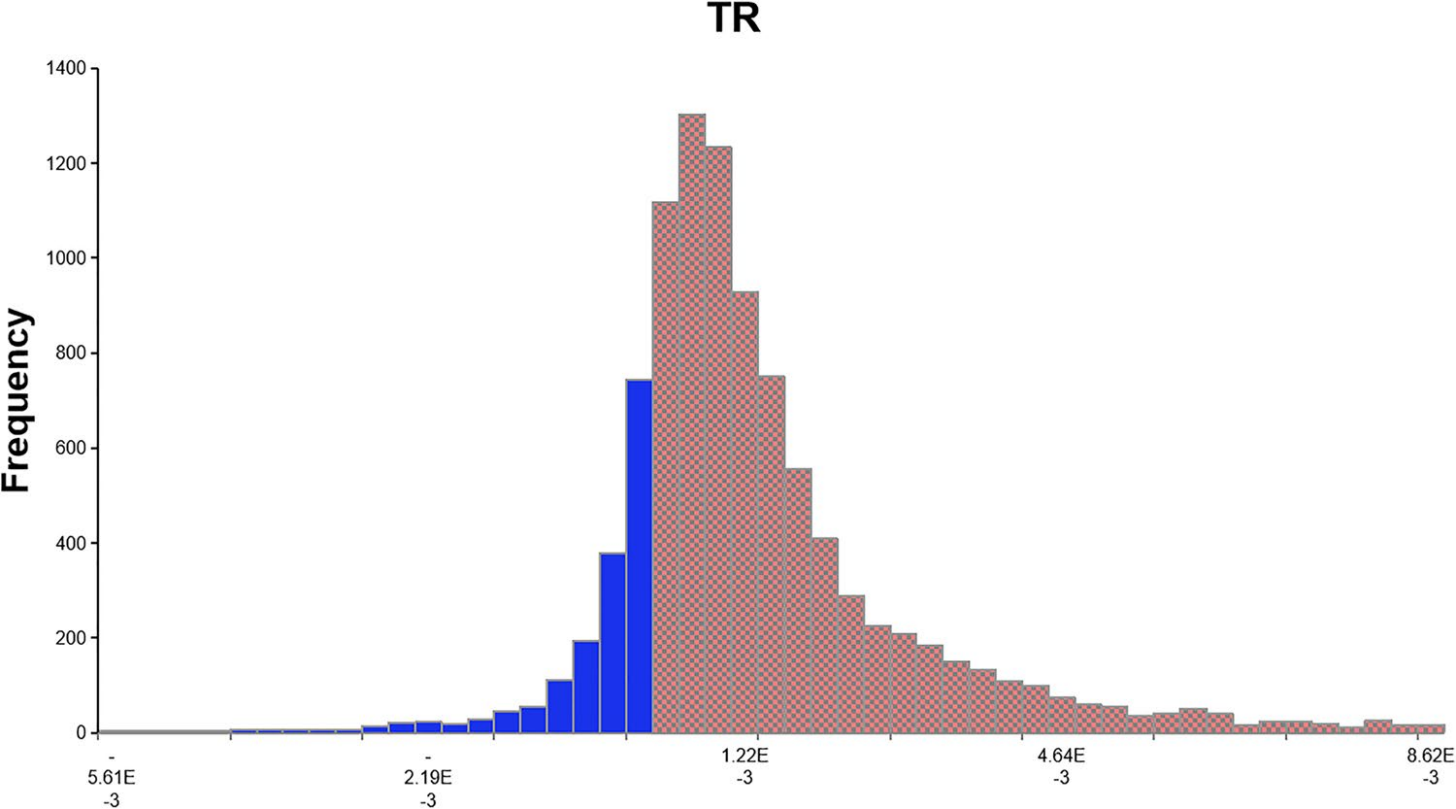
Cumulative distribution of carcinogenic risk (R) of total dietary intake of arsenic (red area represents the probability of exceeding the maximum acceptable levels, which is 87.02%)

To determine the carcinogenic risk, the *R* values for drinking water and various foods were fitted to lognormal distributions. The 90th percentile of *R* values for beans, potatoes, and vegetables were 0.00 × 10^−4^, 0.86 × 10^−4^, and 0.48 × 10^−4^, respectively, with none of them exceeding the maximum acceptable level of 1.0 × 10^−4^. According to the probability estimation results, the 10th, 50th, and 90th percentiles of *R* values were 0.00 × 10^−4^, 2.43 × 10^−4^, and 12.21 × 10^−4^ for drinking water; 0.71 × 10^−4^, 2.07 × 10^−4^, and 7.00 × 10^−4^ for meat; 0.00 × 10^−4^, 1.66 × 10^−4^, and 8.56 × 10^−4^ for cereals; 0.00 × 10^−4^, 2.32 × 10^−4^, and 9.15 × 10^−4^ for solanaceous fruits; and 0.05 × 10^−4^, 0.71 × 10^−4^, and 6.21 × 10^−4^ for fruits. Therefore, we can conclude that the intake of the types of food analyzed in the present study by residents leads to varying degrees of carcinogenic risk, and the risk levels are significantly different. Approximately, 87.02% of the local residents had a total risk (TR) exposure value greater than 1.0 × 10^−4^ (Fig. 2), indicating that they experienced unacceptable carcinogenic risk due to the dietary intake of As.

*Arsenic was not detected in beans, so the distribution could not be modeled

#### Sensitivity Analysis

**Fig. 3:**
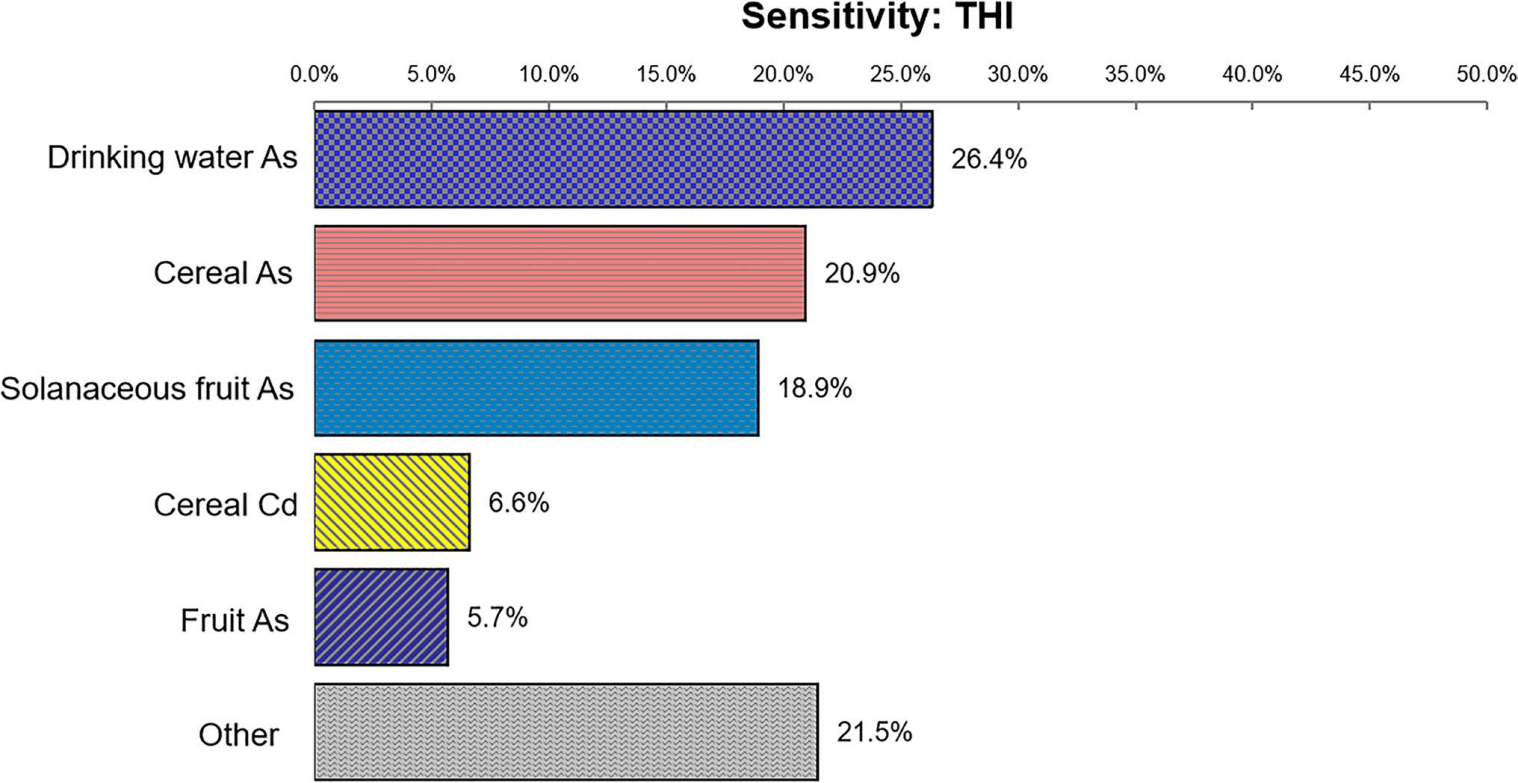
Sensitivity analysis of noncarcinogenic risk from dietary intake of toxic metals

**Fig. 4:**
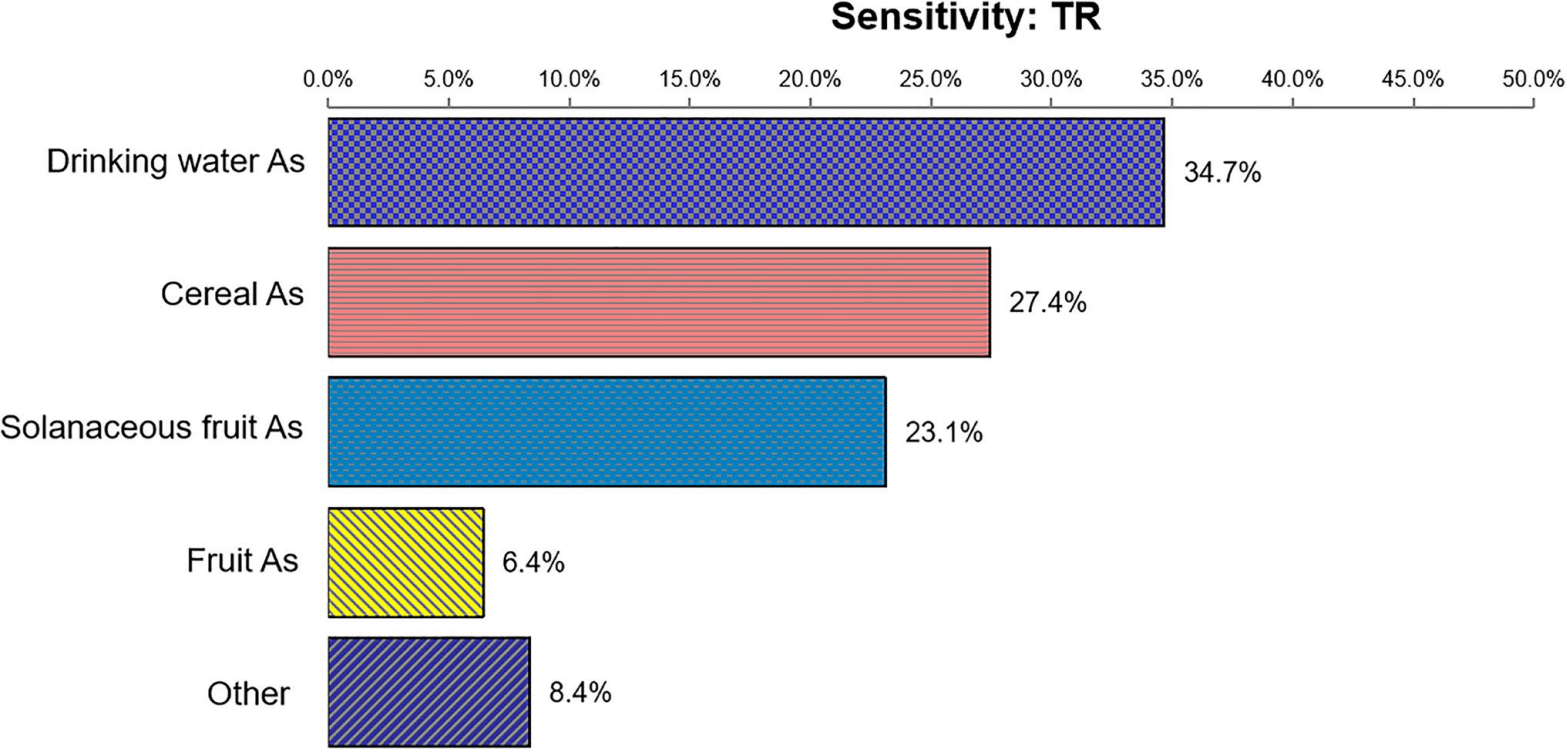
Sensitivity analysis of carcinogenic risk from dietary intake of toxic metals

The results of the sensitivity analysis are shown in Fig. 3 and Fig. 4. For the total noncarcinogenic risk, As in drinking water was the most notable contributor to the health risk, accounting for 26.4%, followed by As in cereals, As in solanaceous fruits, Cd in cereals, and As in fruits, with proportions of 20.9%, 18.9%, 6.6%, and 5.7%, respectively. Regarding the total carcinogenic risk, As in drinking water, cereals, solanaceous fruit, and fruits contributed the most to the output variance, with proportions of 34.7%, 27.4%, 23.1%, and 6.4%, respectively. The results imply that As in drinking water is the most sensitive factor for both noncarcinogenic and carcinogenic risks, and monitoring As concentrations could effectively reduce the health risk it poses to local residents.

## Conclusion

The concentrations of Al, As, Cr, Cd, Cu, Ni, Pb, and Zn in drinking water and seven types of food exceeded the corresponding standard limits at different levels. The most harmful metals, especially Pb, exceeded the standard limits in fruits. The dietary ingestion of these eight toxic metals could pose serious noncarcinogenic risks, with As posing the highest carcinogenic risk to residents in the northern Ningxia region. The As in drinking water was the most sensitive factor affecting health risk results among the various exposure factors evaluated. Therefore, monitoring the concentration of toxic metals in both drinking water and food is necessary. Moreover, the constant monitoring of toxic metals in all food commodities is needed to evaluate whether any potential health risks from heavy metal exposure exist, ensure food safety, and protect residents from food that might pose a risk to their health. However, there are some uncertainties in this study, and the bioavailability of toxic metals in food after being processed and entering the human body was not considered in the assessment. The dietary structure and eating habits of different consumers may also affect their exposure to toxic metals through their diets. A more accurate risk exposure assessment can be carried out in future studies. The results of the present study can serve as an important reference for future research in related fields.

## Data Availability

The datasets used and/or analyzed during the current study available from the corresponding author on reasonable request.
